# The role of molecular chaperonins in warm ischemia and reperfusion injury in the steatotic liver: A proteomic study

**DOI:** 10.1186/1471-2091-13-17

**Published:** 2012-09-10

**Authors:** Venkataswarup Tiriveedhi, Kendra D Conzen, Jane Liaw-Conlin, Gundumi Upadhya, James Malone, R Reid Townsend, Robnet Kerns, Jianluo Jia, Krista Csontos, Sabarinathan Ramachandran, Thallachallour Mohanakumar, Christopher D Anderson, William C Chapman

**Affiliations:** 1 Department of Surgery, Washington University in St. Louis, School of Medicine, St Louis, MO, USA; 2 Department of Medicine, Washington University in St. Louis, School of Medicine, St Louis, MO, USA; 3 Department of Cell Biology and Physiology, Washington University in St. Louis, School of Medicine, St Louis, MO, USA; 4 Department of Pathology and Immunology, Washington University in St. Louis, School of Medicine, St Louis, MO, USA; 5 Department of Surgery, University of Mississippi Medical Center, Jackson, MS, USA; 6 Department of Surgery, Washington University School of Medicine, Box 8109, 3328 CSRB, 660 S. Euclid Ave, St Louis, MO, 63110, USA; 7 Department of Surgery, Washington University School of Medicine, Queeny Tower-6107 660 S. Euclid Ave, St Louis, MO, 63110, USA

**Keywords:** Ischemia repurfusion injury, Two dimensional gel electrophoresis, Mass spectrometry, Liver transplantation, Chaperonins, Endoplasmic reticulum (ER) stress

## Abstract

**Background:**

The molecular basis of the increased susceptibility of steatotic livers to warm ischemia/reperfusion (I/R) injury during transplantation remains undefined. Animal model for warm I/R injury was induced in obese Zucker rats. Lean Zucker rats provided controls. Two dimensional differential gel electrophoresis was performed with liver protein extracts. Protein features with significant abundance ratios (p < 0.01) between the two cohorts were selected and analyzed with HPLC/MS. Proteins were identified by Uniprot database. Interactive protein networks were generated using Ingenuity Pathway Analysis and GRANITE software.

**Results:**

The relative abundance of 105 proteins was observed in warm I/R injury. Functional grouping revealed four categories of importance: molecular chaperones/endoplasmic reticulum (ER) stress, oxidative stress, metabolism, and cell structure. Hypoxia up-regulated 1, calcium binding protein 1, calreticulin, heat shock protein (HSP) 60, HSP-90, and protein disulfide isomerase 3 were chaperonins significantly (p < 0.01) down-regulated and only one chaperonin, HSP-1was significantly upregulated in steatotic liver following I/R.

**Conclusion:**

Down-regulation of the chaperones identified in this analysis may contribute to the increased ER stress and, consequently, apoptosis and necrosis. This study provides an initial platform for future investigation of the role of chaperones and therapeutic targets for increasing the viability of steatotic liver allografts.

## Background

Approximately 6,000 liver transplants are performed annually in the United States (U.S. Scientific Registry and Transplant Recipients 2008). The shortage of available organs, remains a major problem and contributes to a waiting list mortality greater than 110 deaths per 1000 patient years on the waiting list [1]. The current donor shortage is further compounded by the increasing prevalence of hepatic steatosis in potential donors. Fatty infiltration of the liver is now the most common adverse condition in potential donors, with a prevalence of 13-50% in some populations [2,3]. Steatotic livers are more susceptible to ischemia/reperfusion (I/R) injury induced by both warm and cold ischemic periods during transplantation, which directly lead to higher rates of primary nonfunction and delayed graft function [4-6]. Initial primary nonfunction predisposes to three-fold increased incidence of early rejection and graft loss within three months of transplantation [7]. The severity of I/R injury is associated with the degree of fatty infiltration. Severely steatotic livers (>60% steatosis) are contraindicated for liver transplantation [5,6]. Although, moderate steatosis (30-60%) is a relative contraindication for transplantation, however, due to the limited availability of the organs, these allografts are also being used with greater frequency in several transplant centers [2].

Efforts have been made to understand the molecular mechanisms responsible for poor graft function in steatotic livers. However, research to date has been limited by focus on individual pathways or molecules. Given the numerous intracellular changes which are likely to occur, a global molecular approach is required to identify mediators of injury. Proteomics provides one approach to identify and study potential biomarkers and mediators and offers the advantage of elucidating overall patterns of injury-induced changes at the protein level, including both changes in relative protein abundance and post-translational modifications of proteins [8,9]. Proteomics has been successfully employed in the study of various liver injury models and models of ischemia/reperfusion (I/R) [10-15]. High levels of oxidative stress and decreased levels of antioxidant proteins have been implicated as important mediators of injury due to ethanol-induced steatosis [16]. Steatosis has been implicated to induce changes in the mitochondrial proteome [17]. The I/R injury in other organs is shown to increase mitochondrial permeability, contributing to activation of apoptotic pathways [18]. Studies have demonstrated that proteins and enzymes involved in lipid and energy metabolism, redox signaling and oxidative stress responses were affected during cold and warm I/R phases in transplantation [19]. Despite research into the mechanisms of injury in liver I/R and in steatosis, a comprehensive study on the proteome in liver subjected to I/R has not yet been studied. Therefore, we performed a proteomic analysis on fatty rat livers following a period of warm ischemia to identify potential mediators of ischemia/reperfusion injury.

## Methods

### Animals

Male obese (n = 3) and lean (control group, n = 3) Zucker rats were purchased from Harlan Laboratories (Indianapolis, IN). Upon arrival at the pathogen-free barrier facility at Washington University in St. Louis, rats weighed 280 to 310 grams and were 8–11 weeks old. Animals had access to standard chow ad libitum and were maintained on a 12 h light-dark cycle. Animal protocols were approved by the Animal Studies Committee of Washington University in St. Louis and experiments were performed in accordance with the National Institutes of Health Guidelines for the Use of Laboratory Animals.

### Liver ischemia

A model of selective (70%) hepatic warm ischemia was used. Anesthesia was induced with 3% inhaled isoflurane and maintained with 1.5 - 2% isoflurane. Animals were placed on a warming table and the abdominal wall was shaved and sterilized with betadine. Laparotomy was performed through a midline incision. The portal triad was dissected free of surrounding tissue. Portal vein and hepatic artery branches to the left and median hepatic lobes were identified and occluded with a microvascular clamp for 45 min (Fine Science Tools, Foster City, CA). This method of partial hepatic ischemia prevents mesenteric venous congestion and permits decompression of the gut through the right hepatic vessels. Observed blanching of the left and median hepatic lobes confirmed appropriate placement of the clamp. The abdomen was covered with saline-moistened gauze during ischemia. Upon portal clamp removal, evidence of reperfusion was observed through immediate color change of the left and median lobes. The abdominal wall was closed with a running 4–0 monofilament suture. After 1 h of reperfusion, a median lobectomy was performed by ligation of the median lobe portal pedicle with a 4–0 silk suture. The left lobe was removed in a similar manner after two hours of reperfusion. All tissue samples were snap frozen in liquid nitrogen and stored at-80°C until processing.

### Sample preparation

The sample preparation for two-dimensional differential gel electrophoresis was performed with liver protein extracts as previously described [20]. Individual pieces of frozen liver tissue (approximately 1 cm^2^) from lean or obese animals were covered with liquid nitrogen in a pre-cooled Biopulverizer (Biospec Products Inc., Bartsville, OK) and crushed to a fine powder. Each sample was solubilized in lysis buffer (Tris-HCl pH 8.5 (30 mM), 7 M urea, 2 M thiourea, and 4% CHAPS), and the total protein content was determined using the Advanced Protein Assay (Cytoskeleton, Inc.). A total pool was generated using equal amounts of each pool to represent all proteins found in the study. An aliquot containing 50 μg of protein from each sample was diluted to 50μL with lysis buffer (Tris-HCl pH 8.5 (30 mM), 7 M urea, 2 M thiourea, and 4% CHAPS) and labeled with 400 pmol of charge-matched cyanine dyes (GE health care, Pittsburgh, PA) 3-[(4-carboxymethyl) phenylmethyl]-3-ethyloxacarbocyanine halide *N*-hydroxysuccinimidyl ester (Cy2,) for lean, or 1-(5-carboxypentyl)-10-methylindodicarbocyanine halide *N*-hydroxysuccinimidyl ester (Cy5) for obese. All chemicals unless otherwise specified were obtained from Sigma-Aldrich (St. Louis, MO). The total pool sample was labeled using 1-(5-carboxypentyl)-10-propylindocarbocyanine halide *N-*hydroxysuccinimidyl ester (Cy3) for combined protein pool as internal standard for intergel comparison. The pool consisted of all 3 samples and was prepared by combining 50 μg of each individual sample. All labeling reactions were carried out for 45 min at 4°C and protected from light. The reaction was quenched with 10 nmol of lysine for 10 min (4°C, protected from light). Labeled lean and obese samples were combined with a total pool aliquot and the resulting mixture was diluted by addition of 300 mL of rehydration buffer (7 M urea/2 M thiourea, 4% CHAPS, 0.5% v/v ampholytes (pH 3–10)) [21].

### Two dimensional electrophoresis (2-de) and imaging

Each combined tripartite-labeled sample (450 μL final volume) was rehydrated into 24 cm, 3–10 NL IPG strips (GE Healthcare, Piscataway, NJ) under low voltage (100 V) for 12 h, followed by isoelectric focusing (IEF) using a Protean IEF cell (Bio-Rad Labs, Hercules, CA) for a total of 65.5 kVh (using a three-step voltage protocol: 500 V and held for 500 Vh, 1000 V and held for 1000 Vh, 8000 V, and held for 64,000 Vh). After focusing, proteins were reduced by placing the IPG strips in 10 mL of equilibration buffer (10 mL, 50 mM Tris (pH 8.8), 6 M urea, 30% glycerol, 2% SDS, bromophenol blue) containing freshly prepared DTT (50 mg) for 15 min at room temperature. The proteins were then alkylated by adding iodoacetamide (600 mg in 10 mL of equilibration buffer). IPG strips were then rinsed with 1X SDS running buffer (25 mM Tris, 192 mM glycine, 0.1% SDS) and layered on 10-20% polyacrylamide gels and sealed with agarose (1% w/v in 16x running buffer). Commercially prepared gels (Jule, Inc., Milford, CT) were cast between low-fluorescence glass plates using bind-silane (GE Healthcare, Piscataway, NJ) to attach the gel to one plate as *per* the manufacturer’s instructions. Second-dimension SDS-PAGE separation was carried out on all gels simultaneously using 5 W/gel for the first 15 min followed by 1 W/gel for 17 h with circulating buffer (20°C) in the lower buffer chamber. Images of the labeled proteins in each gel were generated using a Typhoon Imager (GE Healthcare, Piscataway, NJ) and the following excitation/emission wavelengths for each dye (488/520 nm for Cy2, 520/580 nm for Cy3, and 620/670 nm for Cy5). After image generation, the nonsilanized glass plate was removed and the gels were placed in fixing solution (33% ethanol, 7.5% acetic acid) for 2 h. Gels were then rinsed with deionized water (18 MΩ) for 15 min and stored in water-filled, sealed bags at 47°C. All steps were performed to minimize exposure to ambient light.

### Gel image analysis

Protein features with significant abundance ratios (fold-change) between experimental groups (p < 0.01) were selected by gel image analysis (DeCyder v. 6.5) and analyzed with high performance liquid chromatography (HPLC)/mass spectometry (MS). ImageQuant (Molecular Dynamics from GE Healthcare, Piscataway, NJ) software was used to crop the gel images and remove portions of the image corresponding to the IPG strip, the spacers between the gel plates, and any remaining dye front from the electrophoresis. The DeCyder (v. 6.5) DIA (difference in-gel analysis) module was used to identify gel feature boundaries and calculate abundance ratios using a normalization algorithm that was applied as previously described [22-24]. Standard parameters were used to determine boundaries estimating 10,000 features per image. Gel artifacts were removed by software from each gel image using a peak volume filter set at 10,000. Additional gel artifacts (*e.g.* water spots, dust particles) were excluded manually. Images were compared across multiple gels using the DeCyder BVA (Biological Variation Analysis) module. This analysis matched features in the pool images from each gel, using this sample as a quantitative reference for protein features in the remaining images allowing quantitative comparison of features in all images in the experiment. The DeCyder Extended Data Analysis (EDA) module was used to perform *t*-test analysis and generate heatmaps. Matched features with significant differences in abundance ratios (p <0.01) or with abundance ratios > 1.5 (p < 0.05) and 100% gel presence were selected for further analysis.

### Gel digestion, preparation of peptides and mass spectrometric analysis

Selected features were excised robotically (ProPic, Genomic Solutions, Ann Arbor, MI) using a triangulation algorithm implemented with in-house software. Proteins in the gel pieces were digested *in situ* with trypsin [25], and stored at −80°C until analyzed by mass spectrometry. Samples were analyzed using a nanoflow (200 nl/min) pulse-free liquid chromatograph, interfaced to a quadrupole time-of-flight mass spectrometer (Q-STAR XL, Applied Biosystems, Foster City, CA) using a PicoView system (New Objective, Woburn, MA), or nano-reversed-phase HPLC interfaced to an electrospray-linear ion trap-Fourier transform ion cyclotron mass spectrometer (LTQ-FT, Thermo-Finnigan, Ringoes, NJ) operated as previously described [26]. The MS and MS/MS data were collected in the profile mode. The “raw” files were processed using MASCOT Distiller, version 2.1.1.0 (Matrix Science, Oxford, U.K.) and searched using MASCOT version 2.3.02 against the UniProt Rat database (downloaded November 21, 2011) with 41, 601 entries (Additional file 1: Table S1). The resulting DAT files were imported into Scaffold, ver. 3.5.1 (Proteome Software, Portland, OR) to identify proteins with greater than or equal to 95% confidence and to determine the spectral counts for each protein. The detailed mass spectrometry and peptide data are summarized in Additional file 1: Table S1.

### Network and pathway analysis

Proteins identified from the Uniprot database were imported into Ingenuity Pathway Analysis (IPA) software, version 6.3 (Ingenuity Systems,Inc., Redwood city, CA) to generate interactive protein networks. Duplicate GI numbers were manually removed prior to loading. GI numbers were loaded into IPA; proteins not recognized by IPA were removed from analysis. A network analysis was performed with limitations set at 35 proteins; direct and indirect relationships were permitted. Core analysis of the protein set generated, producing 9 interactome networks.

Gene networks were created by submitting gene symbol lists to the GRANITE web application (Gene RelAtional Network of InTeracting Elements), a tool to generate gene relational networks (http://bioinformatics.wustl.edu/webTools/GraniteGeneSearch.do). The “enhanced” GRANITE function was used to discover genes not present in but are targets of or interact with submitted gene sets. Gene sets combined with any discovered genes were reloaded into GRANITE to complete connections from discovered genes to genes in the original list. Raw and filtered GRANITE output was directly loaded into Cytoscape for network integeration and visualization [27].

## Results

### Identification of protein targets in warm I/R injury

**Figure 1:**
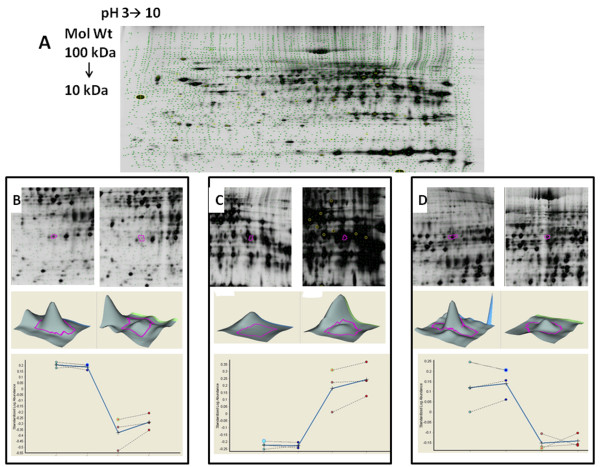
**(A) Isoelectric focusing 2D gel (pH 3–10).** Soluble protein (500 μg) was loaded onto the gel. A representative gel from the total protein collected from of 2 hrs reperfusion injury of liver collected from the obese rat is shown; (B-D) Representative features with abundance as analyzed by three-dimensional simulation and identified by Decyder v6.5 with at least 2 fold abundance.

To analyze the differences in the proteome leading to warm I/R injury in steatotic livers, total protein was collected from lean and obese Zucker rats. A 50 μg protein sample individually collected from lean and obese rats subjected to I/R injury were labeled with cyanine dye Cy2 and Cy5, respectively, and subjected to 2-DE with an IEF range of pH 3 to 10. The proteome of lean and obese rats were compared following 1 and 2 h of reperfusion (Figure 1A). The total protein pool of 25 μg was treated with cyanine dye Cy3 and run on 2-DE as internal standard to compare between intergel variability. The differential in gel analysis (DIA) module for software Decyder v 6.5 (Amersham) was used to calculate the protein spot volumes and normalized volume ratio for each differentially labeled co migrated protein (Figure 1). The values of 2 standard deviations of the mean volume ratios were between 1.3 and 1.7, and only features with greater than 1.5 fold changes in volume in both gels per time point were defined as altered. As shown in lower panel of Figure 1B-D, there is no significant difference between the 1 and 2 hr reperfusion of lean rats, and similarly there is no significant difference between the 1 and 2 hr reperfusion of obese rats.

**Figure 2:**
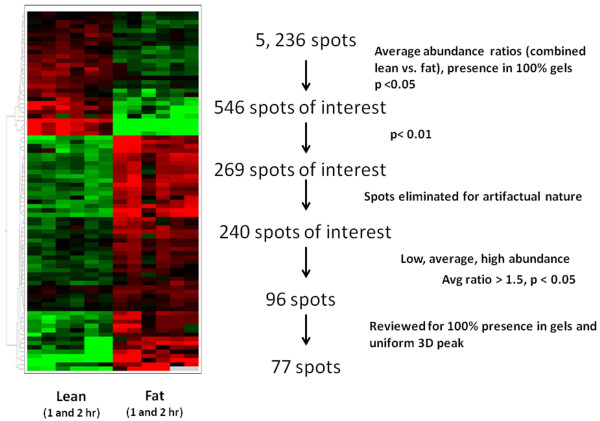
(A) Heat map generated from 84 protein features selected on 2-DE for analysis; (B) Rationale adopted in identifying the features of interest on 2-DE.

A heat map generated from the DeCyder Extended Data Analysis (EDA) module (Figure 2A) was utilized for hierarchical clustering and additional statistical analysis. As shown in Figure 2B, an initial analysis showed 5,236 features on the gels which have a volume ratios (spot frequency/spot volume ratios) variance of at least greater than 1.5. This ratio was taken as a threshold value above or below which spot volume observed in the analytical gels were considered significant. The standardized abundances of all features matched protein features were compared across four analytical protein gels (1 and 2 hrs reperfusion of lean and obese animals), and Students’ *t*-test was performed with the DeCyder biological variance analysis (BVA) module to validate the significance of the detected difference between spot volumes at p < 0.01. A total of 269 features were selected from the initial automated analysis. Manual inspection for artifact elimination (due to streaks and staining contamination), and further statistical analysis, produced 84 features of interest (Additional file 1: Table S1) for feature excision, *in-situ* gel digestion and identification using tandem mass spectrometry with database searching.

### Identification of the differentially regulated proteins by mass spectrometry

The 84 features of interest identified by ANOVA analysis on the 2-DE were excised subjected to in-gel digestion with trypsin, and the peptide pools from each excised feature were analyzed using nano-LC-MS, as described under ‘Materials and Methods’. The database searches were qualified using the Protein Prophet algorithm (95% protein probability) within the SCAFFOLD software. The analysis identified 106 proteins with 453 peptides in the 84 ‘differential’ features (see Additional file 2: Table S2 for peptide sequences and supporting MS data). These lead protein targets were utilized for further analysis to identify the biochemical pathways involved in the I/R injury.

### Differential expression of ER stress related molecular chaperones in steatotic liver after I/R compared to lean liver

**Figure 3:**
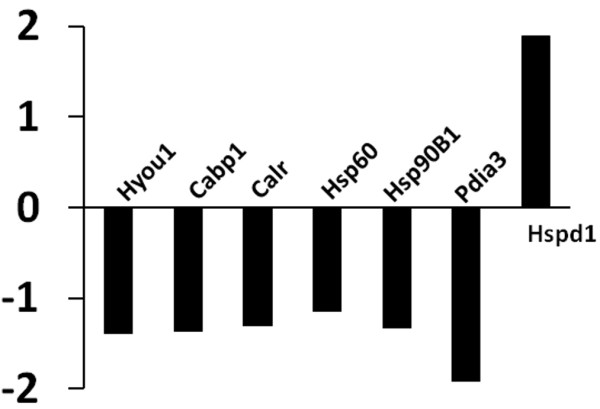
Proteins of chaperonin family which demonstrated significant change in expression profile between lean and obese animals.

No differential gene expression was observed between 1 and 2 h of reperfusion in either the steatotic liver or the lean liver. Therefore, steatotic liver tissue from 1 h and 2 h reperfusion were pooled, and lean liver tissue obtained after 1 or 2 h reperfusion were pooled. All further analysis was performed to compare protein expression between fat and lean liver samples. One hundred four proteins with significant abundance ratios were identified. A PubMed/Medline search was performed for each protein by protein name and corresponding gene ID. Based on published literature in this database, proteins were grouped according to functional category. Functional grouping revealed four categories of importance: molecular chaperones/endoplasmic reticulum (ER) stress, oxidative stress, metabolism, and cell structure (Figure 3). Hypoxia up-regulated 1 (Hyou1, abundance ratio −1.39), calcium binding protein 1 (Cabp1, -1.37), calreticulin (Calr, -1.31), heat shock protein 60 (Hsp60, -1.14), heat shock protein 90 (Hsp90B1, -1.33), and protein disulfide isomerase 3 (Pdia3, -1.92) were chaperonins down-regulated in steatotic liver following I/R. Only one chaperonin, heat shock protein 1 (Hspd1, 1.89), was significantly upregulated.

### Ingenuity pathway analysis identifies four molecular chaperone mediated pathways as the critical targets of I/R injury

**Figure 4:**
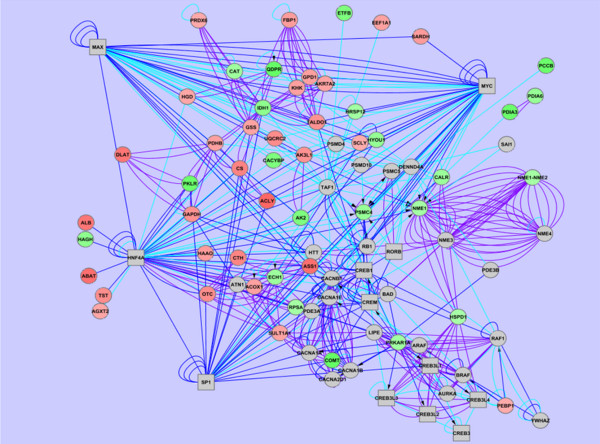
Ingenuity Pathway Analysis was used to define potential relationships of interest between proteins identified in the proteomic analysis.

Ingenuity Pathway Analysis was used to determine relationships of our known significant proteins with each other and other known proteins in the literature which did not show differential expression in our project. IPA generated nine networks, with 106 proteins mapping into at least one of these networks (Figure 4). A network of great significance was that representing cellular assembly and organization, protein degradation, with connections to more than 21 identified proteins with differential expression from the liver samples. This included four of the chaperonins previously identified: Hyou1, Calr, HSP90B1, and Pdia3. These four proteins were indirectly related through interactions with Akt, a well-known serine/threonine kinase, and X-box binding protein-1 (spliced) (XBP-1(s)), a key mediator in one of three ER stress response pathways. A total of 97 proteins were identified in functional or canonical pathways. Not surprisingly, the greatest functional categories and canonical pathways with differential profiles between steatotic and lean liver were those involving amino acid metabolism and small molecule biochemistry.

## Discussion

The recent genomic revolution has resulted in a paradigm drift in the comprehensive analysis of biological processes and biochemical regulators. A continuous body of literature strongly indicating a non-predictive correlation between mRNA and protein level expression [28], along with posttranscriptional modifications leading to control in the rate of synthesis and half-life of proteins place [29] an increased importance on the direct measurement of the protein expression by proteomic techniques to analyze the biochemical signaling pathways. In this report using various proteomics based approaches we determine the key role of regulatory molecular chaperonins in the I/R injury in the setting of liver steatosis.

The demand for liver allografts remains a challenging problem for transplant centers in the US and across the globe, especially with the increasing prevalence of hepatic steatosis in the donor population [30]. Attenuation of steatosis-associated ischemia/reperfusion injury is an important strategy for reducing allograft shortages. Proteomic analysis is an important tool for large-scale analysis of cellular changes induced through various injury models, including that of hepatic ischemia . This particular analysis revealed that the proteome profile of steatotic liver following a period of warm ischemia/reperfusion significantly differs from that of lean liver. Some functional groups, such as those involved with amino acid metabolism, fatty acid metabolism, and cellular structure, are expected to be significantly different in steatotic liver, regardless of I/R injury. A novel finding, however, was the significant down-regulation of multiple molecular chaperones. Chaperonins have important roles in protein folding and structure [31]. Disruptions in cellular homeostasis can cause perturbations in protein processing, resulting in an accumulation of unfolded proteins. This triggers the “unfolded protein response” (UPR), or endoplasmic reticulum (ER stress) response, a response to cellular stress which can lead to adaptation or trigger apoptotic pathways [32]. Chaperonins reduce ER stress by stabilizing proteins which become unfolded during periods of cellular stress (*e.g.*, I/R). Pdia3 encodes ERp57, a known molecular chaperone with well-established interactions with calreticulin and their role in antigen-processing/loading of MHC class I molecules [33,34]. ERp57 is also known to regulate Grp78, a key molecular chaperone in initiating one of three ER stress signaling cascades [35]. The chaperonin subset of proteins, with these molecules in particular, represent potential therapeutic targets for mitigating I/R injury.

The Akt pathway, a serine/threonine kinase, has been shown to play an important role in various types of ischemia/reperfusion. Although it did not show significant differential expression at the reperfusion time points analyzed in our study, Akt is indirectly related to multiple chaperonins which do play a significant role in protein folding and processing, and have implicated roles in endoplasmic reticulum stress in other models. Akt has also been shown to play a role in ER stress. Given these relationships which we have identified, it is likely that ER stress plays a role in the greater injury sustained by steatotic liver following intervals of ischemia/reperfusion [36]. Also, certain key mediators of the unfolded protein response (Grp78, IRE-1a, XBP-1(s), ATF4, and CHOP) are shown to be upregulated in both a murine model of hepatic warm I/R and a rat liver transplant model [37].

The potential role of chaperones as drug targets has been well established. Previous studies in our laboratory using a chaperones targeting small chemical molecule, taurine-conjugated ursodeoxycholic acid (TUDCA), has shown to decrease ER stress response and decrease ischemia repurfusion injury in animal models of steatotic liver allografts [38]. The role of chaperones in ischemia injury has also been established in other transplant models such as cardiac myocytes [39]. Not just limited to transplant settings, but in other disease states such as malaria, chaperones are considered as important drug targets [40]. However, in our initial study along with chaperones/ER stress protein group we have also identified other protein groups such as oxidative stress, metabolism, and cell structure. Although, it is currently unclear of their potential role in drug targeting these protein groups in ameliorating I/R injury and enhancing survival of steatotic liver allografts, future research on these protein groups could offer novel molecular targets.

Certain inherent limitations with 2-DE based proteomics approaches associated with artifacts and database subset mapping in identifying exact target proteins [41] warrants further direct cell based functional studies to validate our current findings. Lean liver tissue served as the comparison, or control, for steatotic liver subjected to warm ischemia and a short period of reperfusion; however, it might be revealing to compare these samples to liver tissue not subject to I/R. Samples obtained at additional reperfusion times would help establish a trend in protein expression profiles. In spite of limitations we still consider our current study to add new insights and further research to study the functional basis and potential chaperonin-based therapeutic strategies to enhance long term survival of the liver allografts.

## Conclusion

In conclusion, using a broad-based proteome analysis, we identified key differences between ischemic responses in steatotic and lean livers. Down-regulation of the chaperonins identified in this analysis may contribute to the increased levels of endoplasmic reticulum stress and, consequently, apoptosis and necrosis observed in steatotic livers compared to lean livers. The proteins identified in this report, provide an initial platform for investigating the role of molecular chaperones in I/R injury in fatty liver and provide potential therapeutic targets for increasing viability of steatotic liver allografts.

## Competing interests

The authors declare they have no competing interests.

## Authors’ contributions

VT participated in the research design, data analysis and writing of the paper; KDC participated in research design performance of research, data analysis and writing of the paper; JL-C and GU participated in the performance of the research; JM, RRT and RK participated in the bioinfomatic analysis and writing of paper; JJ and KC participated in the research design and performance of research; SR, TM and CDA participated in the research design and writing of the paper; WCC is the PI of this project and participated in the research design, data analysis and writing of paper. All authors have read and approved the final manuscript.

## Supplementary Material

Additional file 1 **Table S1.** Identified proteins of interest based on features of interest from 2-DE analysis. Click here for file

Additional file 2 **Table S2.** Target proteins predicted from the excised spot of interest on 2-DE and further analyzed on mass spectrometry and database search by MASCOT. Click here for file
